# A New Sulfated α-Ionone Glycoside from *Sonchus erzincanicus* Matthews

**DOI:** 10.3390/molecules15042593

**Published:** 2010-04-12

**Authors:** Ufuk Ozgen, Handan Sevindik, Cavit Kazaz, Demet Yigit, Ali Kandemir, Hasan Secen, Ihsan Calis

**Affiliations:** 1 Department of Pharmacognosy, Faculty of Pharmacy, Atatürk University, 25240 Erzurum, Turkey; E-Mail: sevindikhandan@yahoo.com (H.S.); 2 Department of Chemistry, Faculty of Sciences, Atatürk University, 25240 Erzurum, Turkey; E-Mails: ckazaz@atauni.edu.tr (C.K.); hsecen@atauni.edu.tr (H.S.); 3 Department of Biology, Faculty of Education, Erzincan University, 24030 Erzincan, Turkey; E-Mails: dyigit@hotmail.com (D.Y.); kandemir_a@hotmail.com (A.K.); 4 Department of Pharmacognosy, Faculty of Pharmacy, Hacettepe University, 06100 Sıhhıye, Ankara, Turkey; E-Mails: icalis@hacettepe.edu.tr (I.C.)

**Keywords:** asteraceae, flavonoids, α-ionone glycoside, *Sonchus erzincanicus*

## Abstract

*Sonchus erzincanicus* (Asteraceae) is an endemic species in Turkey, where six *Sonchus* species grow. In this study, a phytochemical study was performed on the aerial parts of the plant. The study describes the isolation and structure elucidation of five flavonoids and two α-ionone glycosides from *S. erzincanicus*. The compounds were isolated using several and repeated chromatographic techniques from ethyl acetate and aqueous phases that were partitioned from a methanol extract obtained from the plant. 5,7,3′,4′-Tetrahydroxy-3-methoxyflavone (1) and quercetin 3-*O*-*β*-D-glucoside (2) were isolated from the ethyl acetate phase, while corchoionoside C 6’-*O*-sulfate (3), corchoionoside C (4), luteolin 7-*O*-glucuronide (5) and luteolin 7-*O*-*β*-D-glucoside (6), apigenin 7-*O*-glucuronide (7) were isolated from the aqueous phase. Corchoionoside C 6’-*O*-sulfate (3), isolated for the first time from a natural source, was a new compound. The structures of the compounds were elucidated by means of ^1^H-NMR, ^13^C-NMR, 2D-NMR (COSY, HMQC, HMBC) and ESI-MS.

## 1. Introduction

The Asteraceae family or Compositae is represented by about 900 genera and 13,000 species [1]. The genus *Sonchus* (Asteraceae) comprises 50 known species worldwide [1], and is represented by six species in the flora of Turkey, one of which, *S. erzincanicus*, is endemic, [2]. *Sonchus* species are variously known as “sütlük”, “kuzu gevreği”, and “eşek marulu” in Turkey [3]. It has been found that some *Sonchus* species contain sesquiterpene lactone glucosides, flavonoids, triterpenes and steroids [4,5]. No phytochemical study has so far been carried out on *S. erzincanicus*. This study describes the isolation and structure elucidation of five flavonoids and two α-ionone glycosides, one being a new compound, from *S. erzincanicus*. 

## 2. Results and Discussion

**Figure 1:**
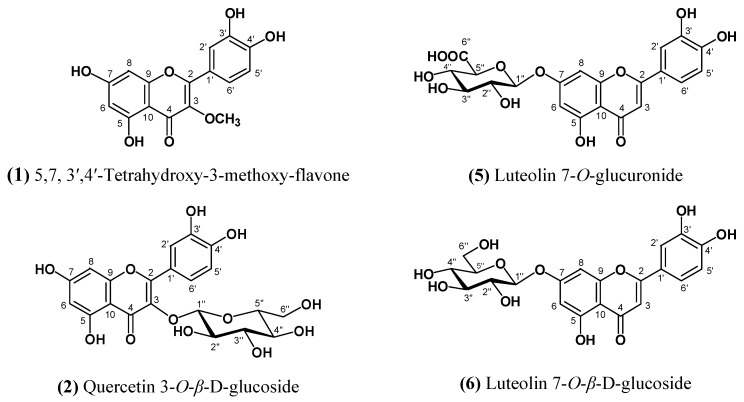

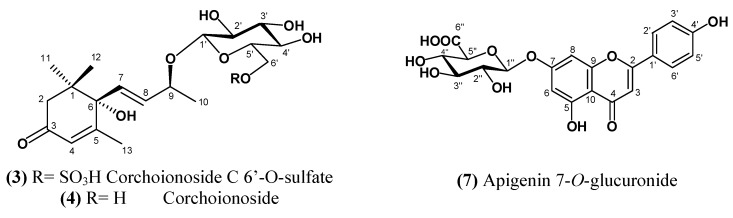
Isolated compounds from *Sonchus erzincanicus*.

In our phytochemical studies on the aerial parts of *Sonchus erzincanicus*, we isolated flavonoids and α-ionone glycosides by using several chromatographic methods. The flavonoids were identified as 5,7,3′,4′-tetrahydroxy-3-methoxyflavone (**1**) [6], quercetin 3-*O*-*β*-D-glucoside (**2**) [7,8], luteolin 7-*O*-glucuronide (**5**) [9], luteolin 7-*O*-*β*-glucoside (**6**) [10] and apigenin 7-*O*-glucuronide (**7**) [11]. Compound **4** was identified as a known α-ionone glycoside, corchoionoside C (**4**) [12]. Compound **3,** corchoionoside C 6’-O-sulfate, was identified as a new natural compound (Figure 1).

The NMR data of compound **3** revealed the presence of a structure similar to that of compound **4**. HRMS spectra of protonated **3** (MH^+^) was 467.1564, which was in agreement with the calculated value: 467.1582. The ESI-MS of compound **3** showed the deprotonated molecule ion peak at m/z 465 [M-H]^-^ and a deprotonated positive ion peak with two added sodium atoms [M-H+2Na]^+^ at 511. The assignments of all proton and carbon resonances (Table 1) were based on 2D NMR (COSY, HETCOR, HMBC) experiments. The anomeric proton signal at δ 4.26 (d, *J* = 7.7 Hz) together with other resonances assigned to the sugar unit having a *β*-glucose moiety. Remaining signals were attributed to the ionone skeleton. All ^1^H-NMR and ^13^C-NMR signals were in agreement with the data given for the structure of corchoionoside C (**4**) except for C-6′ and H_2_-6′ due to esterification at this location [13]. While C-6′ of corchoionoside C (**4**) resonates at δ 61.6 ppm, the same carbon of its sulfate derivative **3** resonates at δ 67.1 ppm due to inductive effect of sulfate ester group. Four diastereomeric reseosides, diastereomers of corchoinoside C, were recently synthesized by Yajima *et al.*: (6S,9S), corchoinoside C; (6S,9R); (6R,9S), (6R,9R). Comparing NMR spectral data of **3** and **4** with the ones of the four reseosides provided clear evidence that both corchoinoside C **4** and corchoionoside C 6’-O-sulfate **3** are in agreement with the structure of (6S,9S) reseoside [14]. Thus, the structure of **3** was established as corchoionoside C 6’-O-sulfate. It is the first time that this compound has been isolated from Nature.

## 3. Experimental

### 3.1. General

^1^H-NMR and ^13^C-NMR spectra were recorded with a Varian Mercury plus spectrometer at 400 and at 100 MHz, respectively. Mass spectra were recorded with Micromass ZQ Mass Spectrometer (Manchester, UK). Sephadex LH-20 (Sigma-Aldrich) and Silica gel (Kiesel gel 60, 0.063-0.2 mm Merck 7734 and 0.040-0.063 mm Merck 9385 and LiChroprep RP-18, 25-40 μm, Merck 9303) were used for column chromatography, while silica gel 60 F_254_ (Merck, 5554) was used for TLC. TLC spots were detected with a UV lamp, spraying 1% Vanillin/H_2_SO_4_ and heated at 120 °C for 1-2 min.

### 3.2. Plant material

The aerial parts of *S. erzincanicus* were collected from Ekşisu (Erzincan Province, Turkey) in 2006 and was identified by Dr. A. Kandemir. A voucher specimen was deposited in the Herbarium of Erzincan University, Faculty of Education (EEFH 7794). 

### 3.3. Extraction and isolation

Dried aerial parts (260 g) of the plant material were extracted by refluxing with methanol (2 L x 3) on a mantle. The methanol extract was concentrated and dried under reduced pressure to give a residue (44.3 g). Methanol extract (44.0 g) was dissolved in H_2_O-MeOH (9:1) and partitioned with chloroform and then ethyl acetate, which were separately concentrated and dried under reduced pressure to give 9.4 g and 0.9 g residues, respectively. The remaining aqueous phase was 32 g. There were too few compounds to isolate and identify in chloroform phase.

The ethyl acetate phase (0.9 g) was subjected to silica gel column chromatography using CHCl_3_-MeOH-H_2_O (80:20:2, 70:30:3, 50:50:5) solvent systems. Fifty nine fractions were collected. Fraction 6 (35.7 mg) gave compound **1** (9 mg) while fractions 18-24 (27 mg) gave compound **2** (15 mg). 

The remaining aqueous phase (32 g) was subjected to reversed phase silica gel column chromatography using 0-100% aqueous MeOH as solvent systems. Fractions were monitored by TLC on silica gel plates and similar fractions were combined to give fraction **A** (Fr. 18-28, 5.5 g), fraction **B** (Fr. 30-40, 334 mg) and fraction **C** (Fr. 45-52, 270 mg).

Fraction A was subjected to silica gel column chromatography with CHCl_3_:MeOH:H_2_O (70:30:3, 65:35:5) solvent system. Fr. 36-47 gave compound **3** (18 mg). 

Fraction B was subjected to a gel chromatography (Sephadex LH-20) eluting with MeOH and 15 fractions were collected. The fractions 2-5 (B1, 157 mg) were further purified by successive column chromatography on silica gel and Sephadex LH-20, respectively, yielding pure **4** (18 mg). The fractions 8-10 (B2, 25 mg) gave compound **5**. 

Fraction C was subjected to a silica gel column chromatography with CHCl_3_-MeOH-H_2_O (70:30:3) and 60 fractions were collected. The fractions 10-14 (C1, 50 mg) were subjected to gel chromatography (Sephadex LH-20) with MeOH to give compound **6** (14 mg). The fractions 41-50 (C2, 41 mg) were subjected to a gel chromatography (Sephadex LH-20) with MeOH to give compound **7** (10 mg).

*Compound* **1**: Yellow powder; ^1^H-NMR (CD_3_OD): δ 7.60 (1H, bs, H-2′), 7.52 (1H, d, H-6′, *J =* 8.4 Hz), 6.89 (1H, d, H-5′, *J =* 8.4 Hz), 6.37 (1H, bs, H-8), 6.18 (1H, bs, H-6), 3.77 (s, OCH_3_); ^13^C-NMR (CD_3_OD): δ 178.8 (C-4), 164.9 (C-7), 161.8 (C-5), 157.2 (C-9), 156.8 (C-2), 148.7 (C-4′), 145.3 (C-3′), 138.3 (C-3), 121.7 (C-1′), 121.1 (C-6′), 115.3 (C-5′), 115.2 (C-2′), 104.6 (C-10), 98.6 (C-6), 93.6 (C-8), 59.3 (OCH_3_). ^1^H-NMR and ^13^C-NMR agree with data given in the literature for 5,7,3′,4′-tetrahydroxy-3-methoxyflavone [6].

*Compound* **2**: Yellow powder; ^1^H-NMR (CD_3_OD): δ 7.70 (1H, d, H-2′, *J =* 1.9 Hz), 7.58 (1H, dd, H-6′, *J =* 8.5 Hz, 1.9 Hz), 6.86 (1H, d, H-5′, *J =* 8.5 Hz), 6.36 (1H, d, H-8, *J =* 2.2 Hz), 6.17 (1H, d, H-6, *J =* 2.2 Hz), 5.22 (1H, d, H-1″, *J =* 7.3 Hz), 3.85-3.30 (6H, sugar protons); ^13^C-NMR (CD_3_OD): δ 178.1 (C-4), 166.2 (C-7), 161.8 (C-5), 157.6 (C-2), 157.4 (C-9), 148.7 (C-4′), 144.7 (C-3′), 134.4 (C-3), 122.0 (C-1′), 121.9 (C-6′), 116.3 (C-5′), 114.8 (C-2′), 104.1 (C-10), 103.2 (C-1″), 99.2 (C-6), 93.8 (C-8), 77.2 (C-5″), 76.9 (C-3″), 74.5 (C-2″), 70.0 (C-4″), 61.4 (C-6″). ^1^H-NMR and ^13^C-NMR agree with data given in the literature for quercetin 3-*O*-*β*-D-glucoside [7,8].

*Compound* **3**: Amorphous colourless solid; [α]_D_^22^ = +38 (c=1, MeOH), ESI-MS (C_19_H_30_O_11_S), m/e: 465 [M-H]^¯^ and 511 [M-H+2Na]^+^. HRMS: calculated for C_19_H_31_O_11_S^+^: 467.1582; found: 467.1564. For ^1^H-NMR (CD_3_OD) and ^13^C-NMR (CD_3_OD). See (Table 1).

*Compound* **4**: Amorphous colourless solid, ESI-MS (C_19_H_30_O_8_), m/e: 409 [M+Na]^+^ and 385 [M-H]^-^, ^1^H-NMR (CD_3_OD): δ 5.97 (1H, d, H-7, *J =* 15.2 Hz), 5.86 (1H, s, H-4), 5.72 (1H, dd, H-8, *J =* 15.5 Hz, *J =* 7.0 Hz), 4.53 (1H, quintet, H-9, *J =* 6.6 Hz), 4.26 (1H, d, H-1′, *J =* 7.7 Hz), 3.85 (1H, dd, H-6_a_′, *J =* 11.9 Hz, *J =* 2.2 Hz), 3.62 (1H, dd, H-6_b_′, *J =* 11.9 Hz, *J =* 6.0 Hz), 3.25-3.34 (sugar protons, overlapped, 4H, H-2′, H-3′, H-4′, H-5′), 2.61 (1H, d, H-2a, *J =* 17.6 Hz), 2.16 (1H, d, H-2b, *J =* 17.6 Hz), 1.94 (3H, bs, H-13), 1.02 (3H, d, H-10, *J =* 8.4 Hz), 0.91 (3H, s, H-11), 0.88 (3H, s, H-12); ^13^C-NMR (CD_3_OD): δ 200.0 (C-3), 166.0 (C-5), 132.6 (C-7), 132.5 (C-8), 125.9 (C-4), 100.1 (C-1′), 77.1 (C-6), 77.0(C-5′), 73.8 (C-3′), 73.4 (C-2′), 70.7 (C-9), 70.5 (C-4′), 61.6 (C-6′), 49.8 (C-2), 41.2 (C-1), 23.5 (C-12), 22.5 (C-11), 22.3 (C-10), 18.4 (C-13). ^1^H-NMR and ^13^C-NMR agree with data given in the literature for corchoionoside C [12].

*Compound* **5**: Yellow powder; ^1^H-NMR (DMSO-*d_6_*): δ 7.40 (1H, d, H-2′, *J =* 2.0 Hz), 7.36 (1H, dd, H-6′, *J =* 8.4 Hz, *J =* 2.0 Hz), 6.84 (1H, d, H-5′, *J =* 8.4 Hz), 6.74 (1H, d, H-8, *J =* 1.9 Hz), 6.69 (1H, s, H-3), 6.39 (1H, d, H-6, *J =* 1.9 Hz), 5.06 (1H, d, H-1″, *J =* 7.3 Hz), 3.60 (1H, d, H-5″, *J =* 9.9 Hz), 3.39-3.14 (m, 3H, sugar protons, overlapped with DMSO-*d_6_* signals); ^13^C-NMR (DMSO-*d*_6_): δ 182.5 (C-4), 172.5 (C-6″), 165.1 (C-2), 163.6 (C-7), 161.7 (C-5), 157.6 (C-9), 150.8 (C-4′), 146.6 (C-3′), 121.7 (C-1′), 119.7 (C-6′), 116.7 (C-5′), 114.1 (C-2′), 105.9 (C-10), 103.6 (C-3), 100.2 (C-1″), 100.2 (C-6), 95.2 (C-8), 77.1 (C-3″), 74.5 (C-5″), 73.6 (C-2″), 72.6 (C-4″). ^1^H-NMR and ^13^C-NMR agree with data given in the literature for luteolin 7-*O*-glucuronide [9].

*Compound* **6**: Yellow powder; ^1^H-NMR (DMSO-*d_6_*): δ 7.42 (1H, bd, H-6′, *J =* 8.8 Hz), 7.40 (1H, bs, H-2′), 6.87 (1H, d, H-5′, *J =* 8.4 Hz), 6.77 (1H, d, H-8, *J =* 1.8 Hz), 6.73 (1H, s, H-3), 6.42 (1H, d, H-6, *J =* 1.8 Hz), 5.06 (1H, d, H-1″, *J =* 7.3 Hz), 3.70-3.15 (6H, sugar protons); ^13^C-NMR (DMSO-*d_6_*): δ 182.5 (C-4), 165.2 (C-2), 163.6 (C-7), 161.8 (C-5), 157.6 (C-9), 151.1 (C-4′), 146.6 (C-3′), 121.7 (C-1′), 119.9 (C-6′), 116.6 (C-5′), 114.1 (C-2′), 106.0 (C-10), 103.7 (C-3), 100.6 (C-1″), 100.2 (C-6), 95.4 (C-8), 77.8 (C-5″), 77.1 (C-3″), 73.8 (C-2″), 70.2 (C-4″), 61.3 (C-6″). ^1^H-NMR and ^13^C-NMR agree with data given in the literature for luteolin 7-*O*-*β*-D-glucoside [10].

*Compound* **7**: Yellow powder; ^1^H-NMR (DMSO-*d_6_*): δ 7.86 (2H, quasi d, H-2′/6′, *J =* 8.8 Hz), 6.88 (2H, quasi d, H-3′/5′, *J =* 8.8 Hz), 6.84 (1H, d, H-8, *J =* 2.0 Hz), 6.62 (1H, s, H-3), 6.49 (1H, d, H-6, *J =* 2.0 Hz), 5.10 (1H, d, H-1″, *J =* 6.6 Hz), 3.90-3.25 (sugar protons, 4H, H-2″, H-3″, H-4″, H-5″); ^13^C-NMR (DMSO-*d_6_*): δ 183.0 (C-4), 175.1 (C-6″), 165.9 (C-2), 163.7 (C-7), 161.8 (2C, C-4′ and C-5), 157.8 (C-9), 128.5 (2C, C-2′/6′), 121.1 (C-1′), 116.4 (2C, C-3′/5′), 106.0 (C-10), 102.4 (C-3), 100.4 (C-1″), 100.2 (C-6), 94.9 (C-8), 76.4 (C-5″), 75.4 (C-3″), 73.4 (C-2″), 72.3 (C-4″). ^1^H-NMR [11] and ^13^C-NMR [15] agree with data given in the literature for apigenin 7-*O*-glucuronide.

## Figures and Tables

**Table 1:** NMR Spectroscopic data for compound **3** (^1^H-NMR: 400 MHz, ^13^C-NMR: 100 MHz).

C/H atom	δC	δH ppm, J (Hz)	HMBC (H→C)
1	41.3		
2	49.6	2.63 d (16.7) 2.16 d (16.7)	C-11, C-12
3	200.2		
4	126.0	5.87 bs	C-6, C-2
5	165.9		
6	78.8		
7	132.5	5.98 d (15.6)	C-9, C-5
8	132.4	5.70 dd (15.6, 7.2)	C-6, C-10
9	73.5	4.50 quintet (6.8)	C-1′, C-7
10	21.0	1.28 d (6.2)	C-8
11	22.3	1.03 s	C-2, C-6
12	23.6	1.01 s	C-2, C-6
13	18.4	1.94 d (1.1)	C-6, C-4
Glucose			
1′	100.1	4.26 d (7.7)	
2′	73.7	3.28-3.36 ^a^	
3′	74.9		
4′	70.3		
5′	76.9		
6′	67.1	4.29 dd, (10.9, 1.8) 4.09 dd (10.9, 5.5)	

^a^ Signal patterns are not clear due to overlapping.

## Supplementary Materials

Supplementary File 1
