# Supplementary material for: A New Sulfated α-Ionone Glycoside from Sonchus erzincanicus Matthews
Source: Molecules. 2010 Apr 12;15(4):2593–9. doi: 10.3390/molecules15042593 (PMC6257313; doi:10.3390/molecules15042593)

Supporting Material

A New Sulfated α-Ionone Glycoside from *Sonchus erzincanicus* Matthews

Ufuk ÖZGEN [1,*], Handan SEVİNDİK [1], Cavit KAZAZ [2], Demet YİĞİT [3], Ali KANDEMİR [3], Hasan SEÇEN [2], İhsan ÇALIŞ [4]

Corchoionoside C sulfate ester (3).

^{1}H-NMR (400 MHz, CD_3OD): δ 5.98 (1H, d, H-7, J=15.6 Hz), 5.87 (1H, bs, H-4), 5.70 (1H, dd, H-8, J=15.6 Hz, J=7.2 Hz), 4.50 (1H, quintet, H-9, J=6.8 Hz), 4.29 (1H, dd, H-6_a', J=10.9 Hz, J=1.8 Hz), 4.26 (1H, d, H-1', J=7.7 Hz), 4.09 (1H, dd, H-6_b', J=10.9 Hz, J=5.5 Hz), 3.28-3.36 (sugar protons, overlapped, 4H, H-2', H-3', H-4', H-5'), 2.63 (1H, d, H-2a, J=16.7 Hz), 2.16 (1H, d, H-2b, J=16.7 Hz), 1.94 (3H, d, H-13, J=1.1 Hz), 1.28 (3H, d, H-10, J=6.2 Hz), 1.03 (3H, s, H-11), 1.01 (3H, s, H-12).

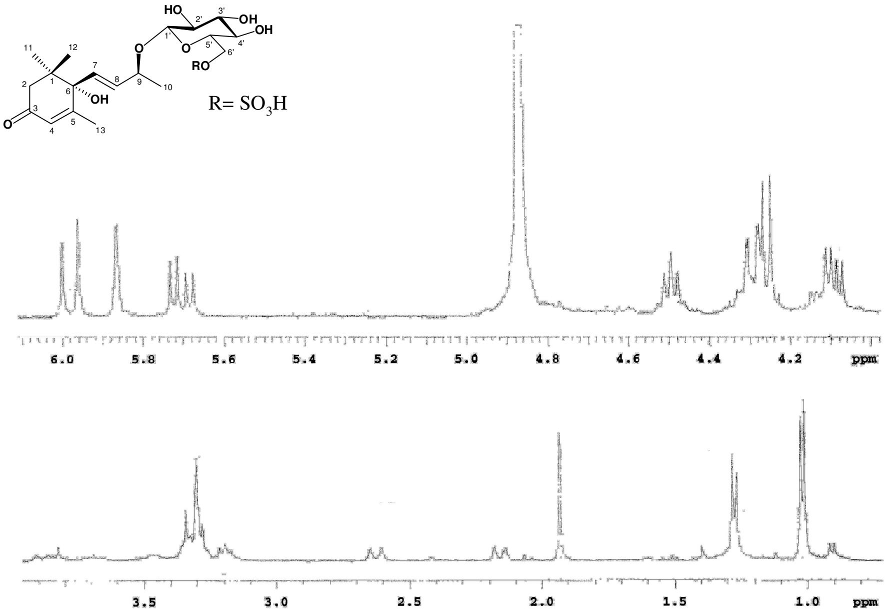

Corchoionoside C sulfate ester (3).

^{13}C-NMR (100 MHz, CD_3OD): δ 200.2 (C-3), 165.9 (C-5), 132.5 (C-7), 132.4 (C-8), 126.0 (C-4), 100.1 (C-1'), 78.8 (C-6), 76.9 (C-5'), 74.9 (C-3'), 73.7 (C-2'), 73.5 (C-9), 70.3 (C-4'), 67.1 (C-6'), 49.6 (C-2), 41.3 (C-1), 23.6 (C-12), 22.3 (C-11), 21.0 (C-10), 18.4 (C-13).

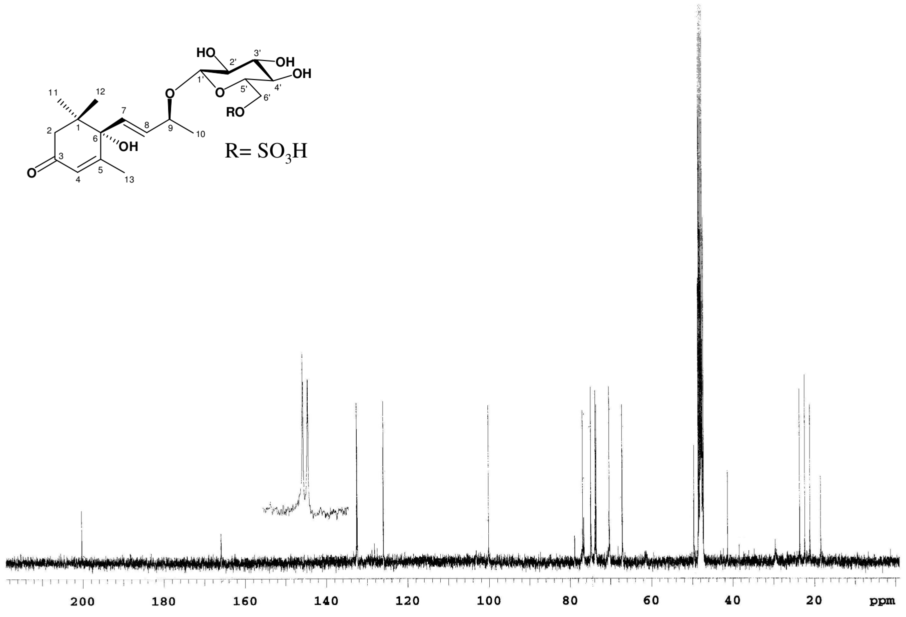

Corchoionoside C sulfate ester (3).

ESI-MS ($C_{19}H_{30}O_{11}S$), m/e: 465 $[M-H]^-$ and 511 $[M-H+2Na]^-$

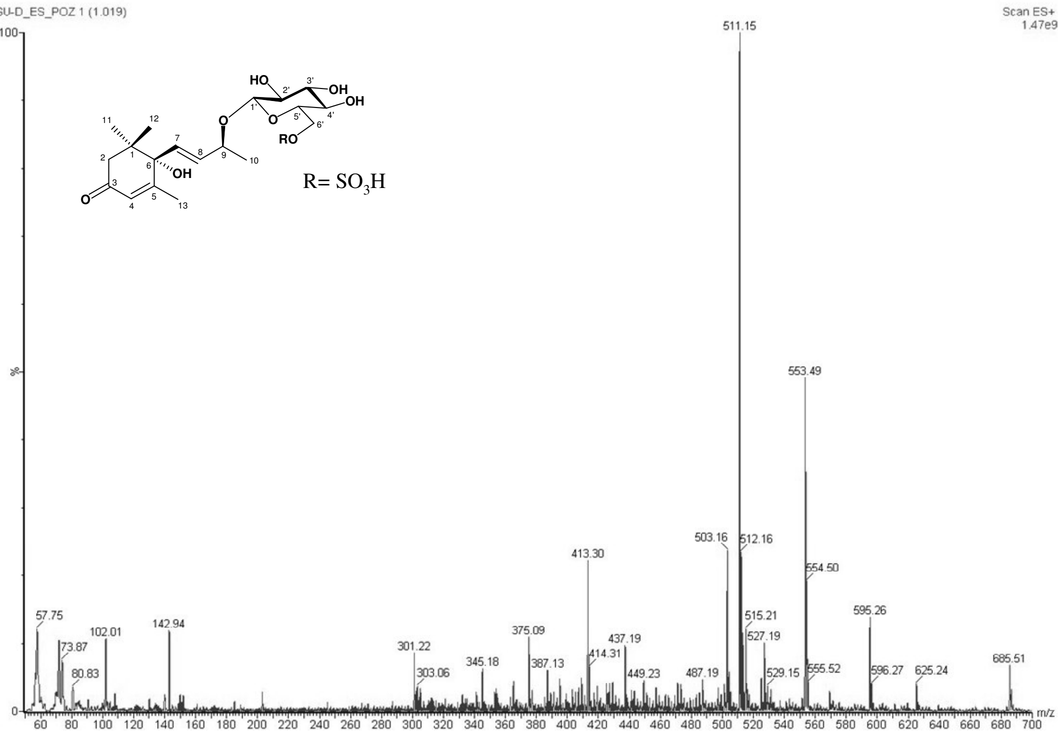

Corchoionoside C sulfate ester (3).

ESI-MS ($C_{19}H_{30}O_{11}S$), m/e: 465 $[M-H]^-$ and 511 $[M-H+2Na]^-$

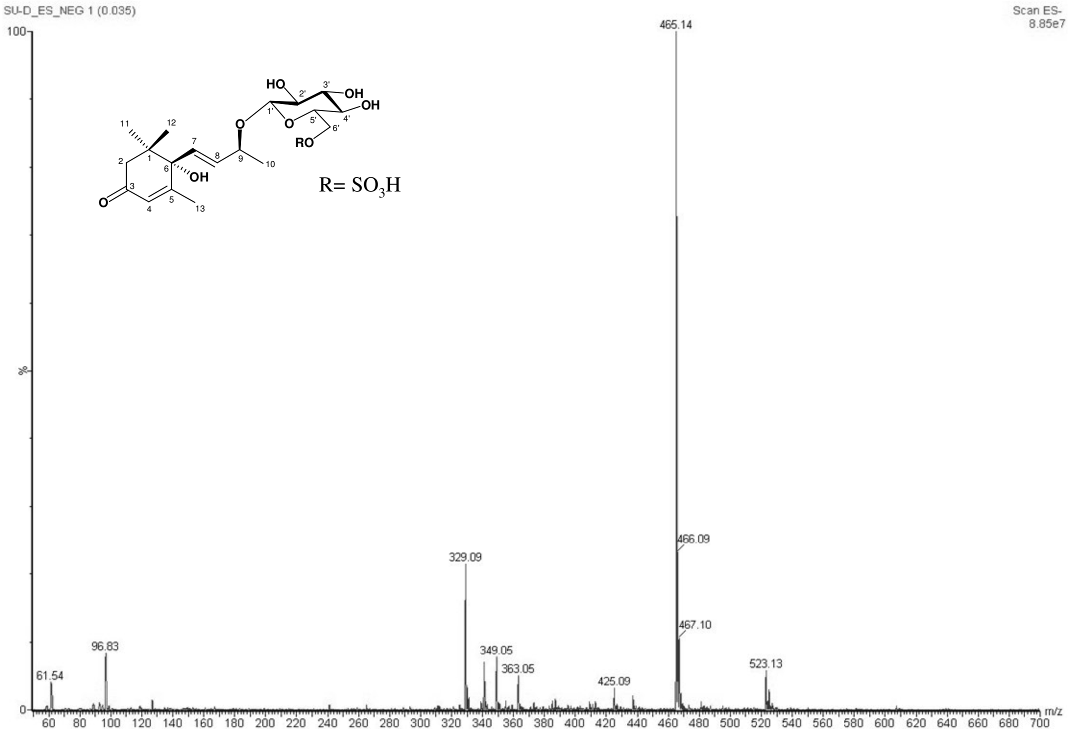

Corchoionoside C sulfate ester (3).

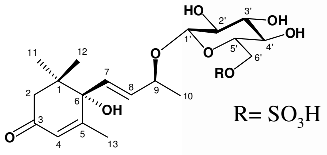

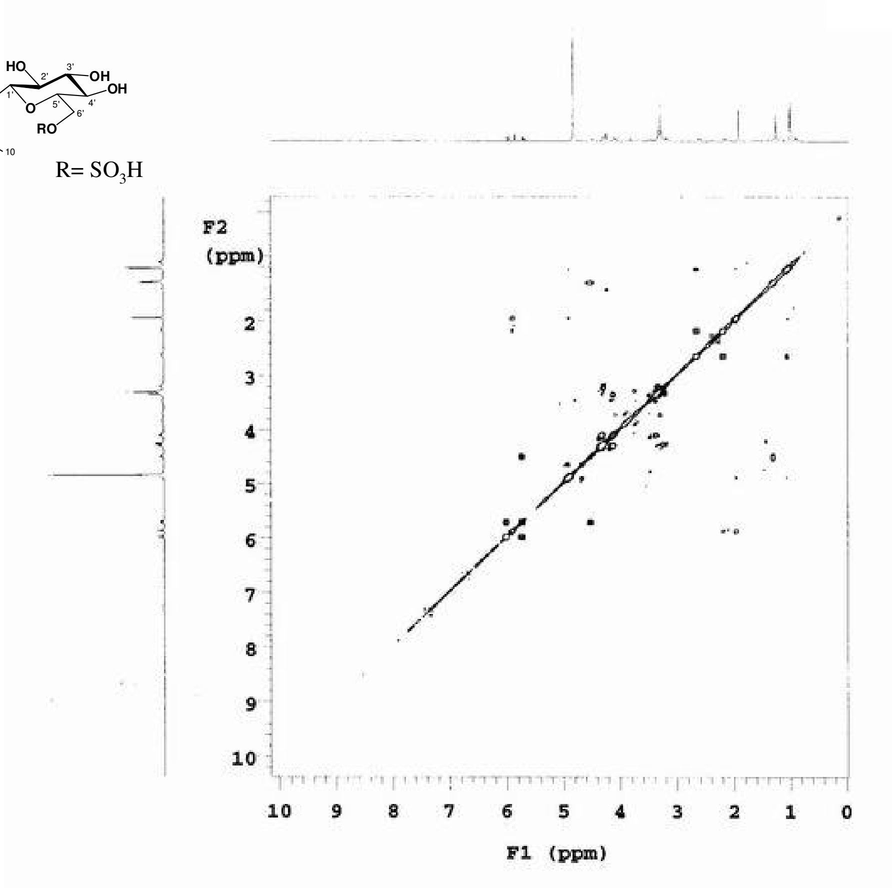

Corchoionoside C sulfate ester (3).

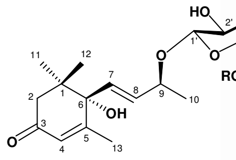

R= SO_3H

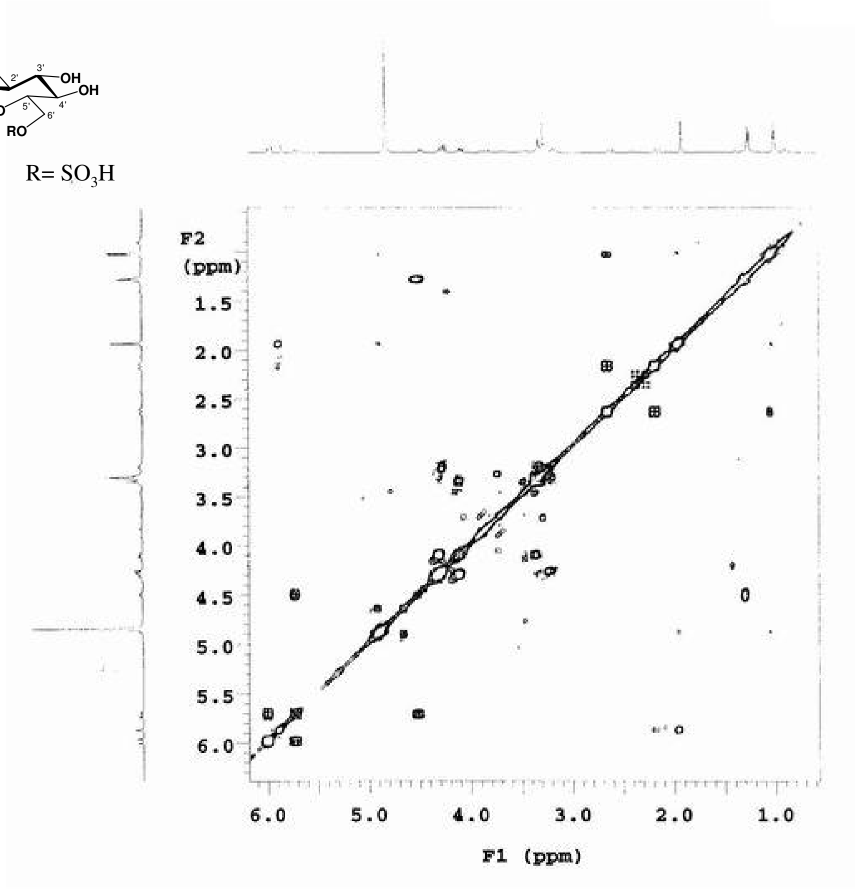

Corchoionoside C sulfate ester (3).

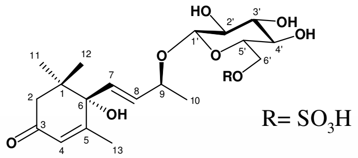

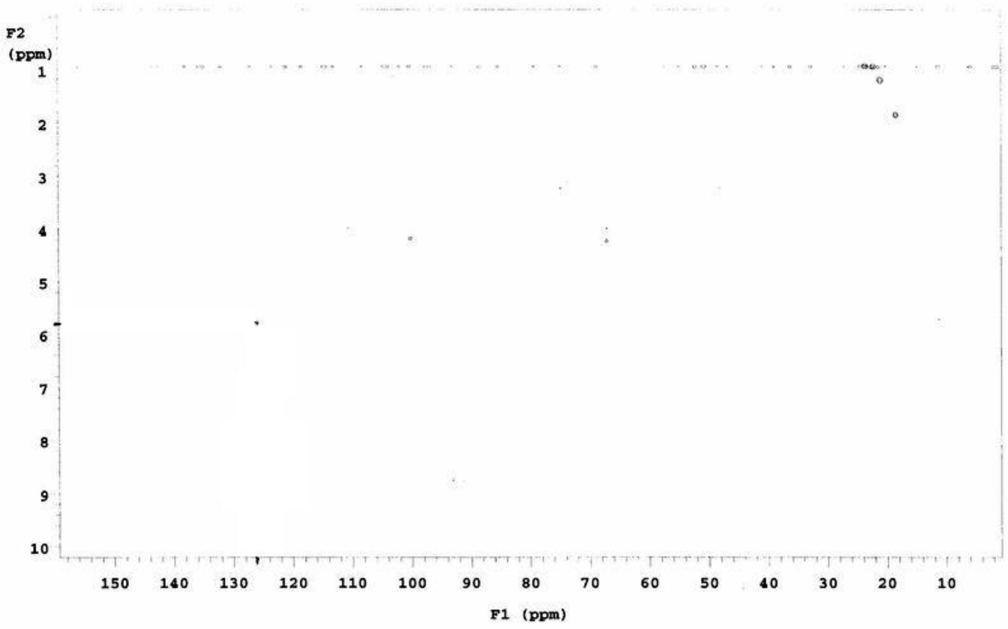

Corchoionoside C sulfate ester (3).

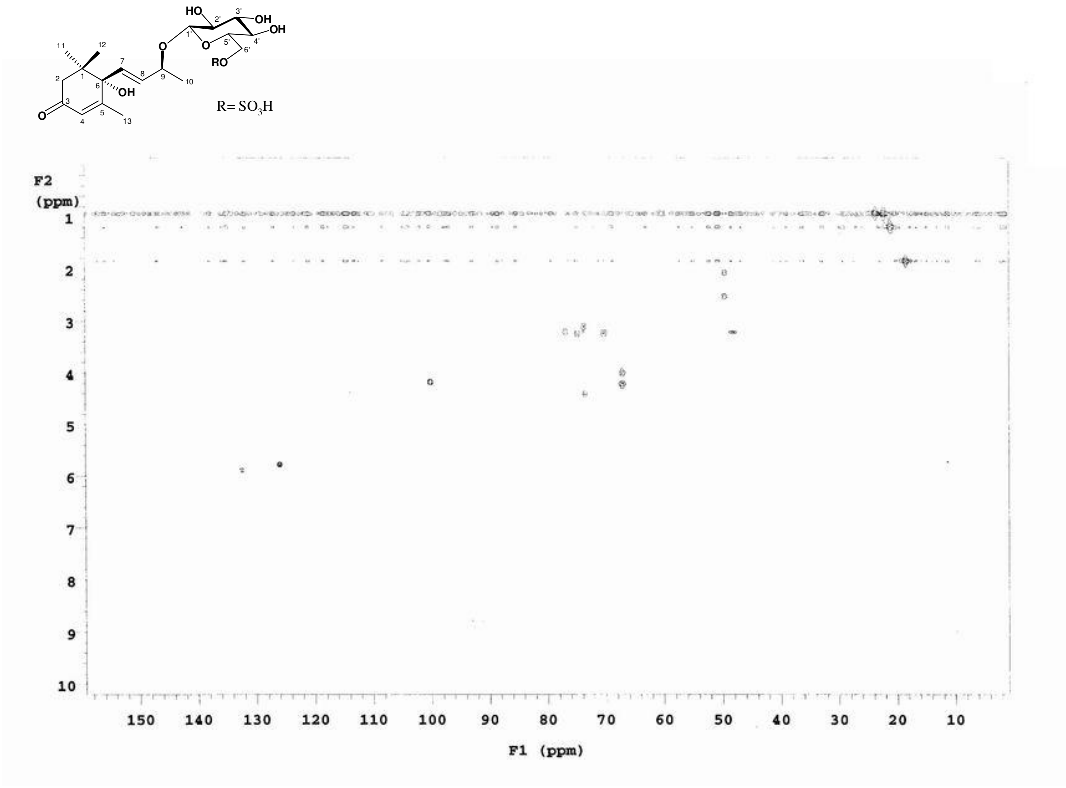

Corchoionoside C sulfate ester (3).

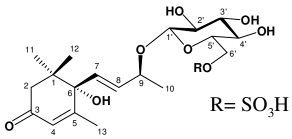

R= SO_3H

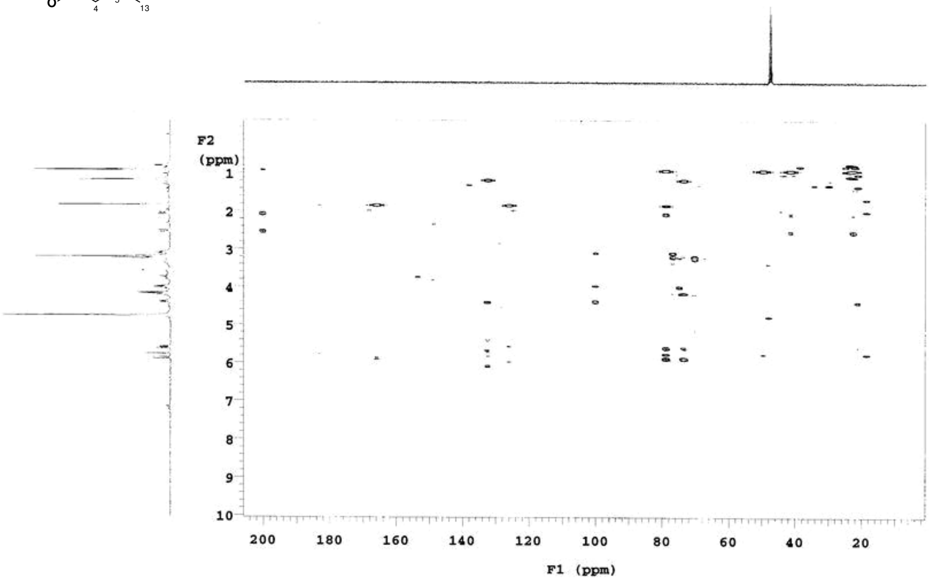

Corchoionoside C sulfate ester (3).

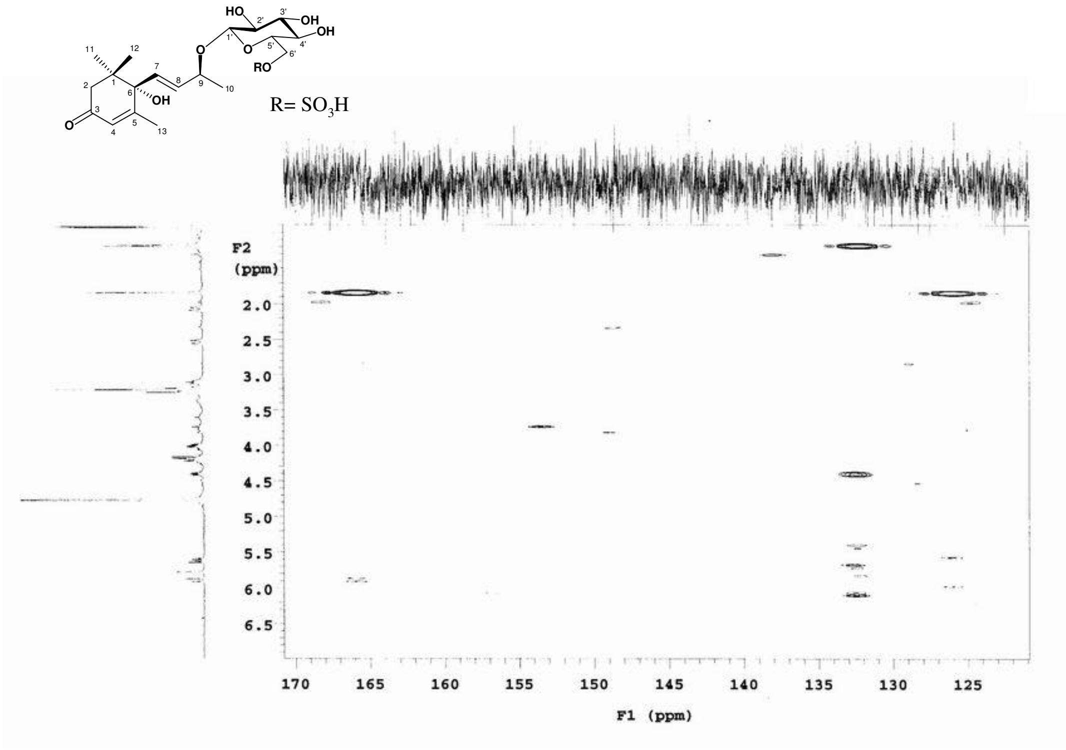

Corchoionoside C sulfate ester (3).

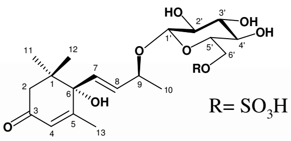

R= SO_3H

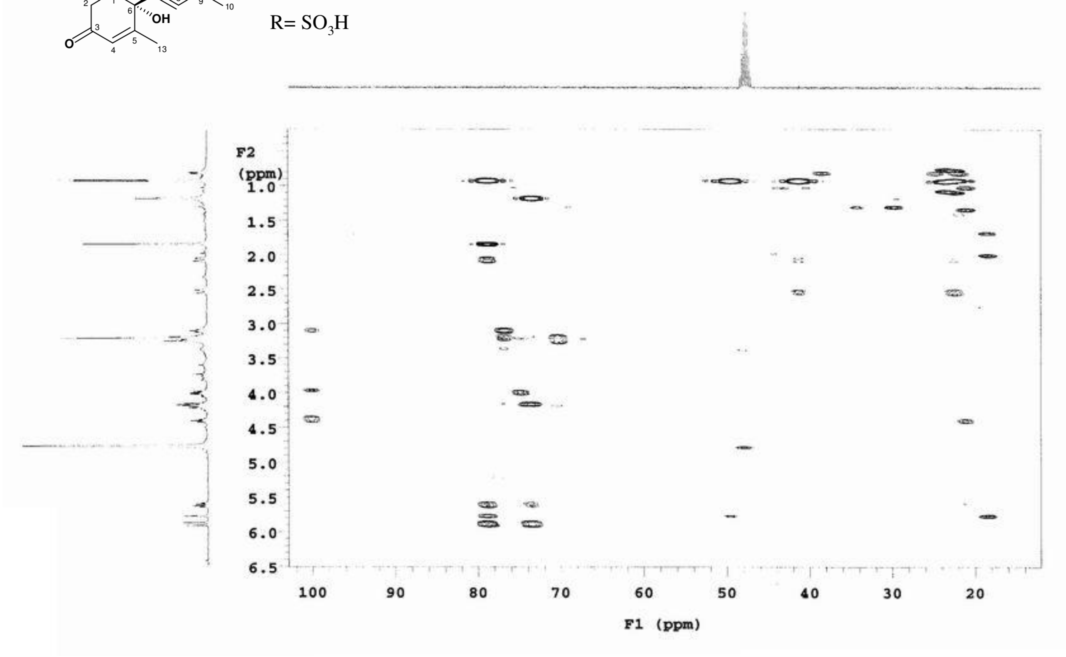

Supplement: Supplementary File 1 [file molecules-15-02593-s001.pdf]
